# Primate lentiviral Nef proteins deregulate T-cell development by multiple mechanisms

**DOI:** 10.1186/1742-4690-10-137

**Published:** 2013-11-15

**Authors:** Anouk Van Nuffel, Kevin K Ariën, Veronique Stove, Michael Schindler, Eduardo O’Neill, Jan Schmökel, Inge Van de Walle, Evelien Naessens, Hanne Vanderstraeten, Kathleen Van Landeghem, Tom Taghon, Kati Pulkkinen, Kalle Saksela, J Victor Garcia, Oliver T Fackler, Frank Kirchhoff, Bruno Verhasselt

**Affiliations:** 1 Department of Clinical Chemistry, Microbiology, and Immunology, Ghent University, Ghent, Belgium; 2 Institute of Molecular Virology, Ulm University Medical Center, Ulm, Germany; 3 Department of Internal Medicine, Division of Infectious Diseases, University of Texas Southwestern Medical Center at Dallas, Dallas, Texas 75390, USA; 4 Department of Virology, Haartman Institute, University of Helsinki and Helsinki University Central Hospital, Helsinki, Finland; 5 Department of Infectious Diseases, Virology, University Hospital Heidelberg, Heidelberg, Germany; 6 Present address: Virology Unit, Institute of Tropical Medicine, Antwerp, Belgium; 7 Present address: Institute of Virology, Helmholtz Zentrum München, München, Germany; 8 Present address: National Center for HIV/AIDS, Viral Hepatitis, STD, and TB Prevention, Centers for Disease Control and Prevention, Atlanta, GA 30333, USA; 9 Present address: Division of Infectious Diseases, Center for AIDS Research, University of North Carolina at Chapel Hill, Chapel Hill, NC 27599-7042, USA

**Keywords:** HIV, SIV, Nef, Thymus, PAK2, CD3, CXCR4

## Abstract

**Background:**

A *nef* gene is present in all primate lentiviral genomes and is important for high viral loads and progression to AIDS in human or experimental macaque hosts of HIV or SIV, respectively. In these hosts, infection of the thymus results in a decreased output of naive T cells that may contribute to the development of immunodeficiency. We have previously shown that HIV-1 subtype B Nef proteins can block human T-cell development. However, the underlying mechanism(s) and the conservation of this Nef function between different groups of HIV and SIV remained to be determined.

**Results:**

We investigated whether reduction of thymic output is a conserved function of highly divergent lentiviral Nef proteins including those from both types of human immunodeficiency viruses (HIV-1 and HIV-2), their direct simian counterparts (SIVcpz, SIVgor and SIVsmm, respectively), and some additional SIV strains. We found that expression of most of these *nef* alleles in thymocyte progenitors impaired T-cell development and reduced thymic output. For HIV-1 Nef, binding to active p21 protein (Cdc42/Rac)-activated kinase (PAK2) was a major determinant of this function. In contrast, selective disruption of PAK2 binding did not eliminate the effect on T-cell development of SIVmac239 Nef, as was shown by expressing mutants in a newly discovered PAK2 activating structural motif (PASM) constituted by residues I117, H121, T218 and Y221, as well as previously described mutants. Rather, down-modulation of cell surface CD3 was sufficient for reduced thymic output by SIVmac Nef, while other functions of SIV Nefs contributed.

**Conclusions:**

Our results indicate that primate lentiviral Nef proteins impair development of thymocyte precursors into T cells in multiple ways. The interaction of HIV-1 Nef with active PAK2 by HIV-1 seem to be most detrimental, and downregulation of CD3 by HIV-2 and most SIV Nef proteins sufficient for reduced thymic output. Since the reduction of thymic output by Nef is a conserved property of divergent lentiviruses, it is likely to be relevant for peripheral T-cell depletion in poorly adapted primate lentiviral infections.

## Background

Humans infected with HIV develop AIDS due to a progressive decline in their lymphocyte numbers and function. An imbalance between production and destruction of peripheral CD4^+^ T cells leads to their progressive decline over time and eventually to AIDS [1,2]. The thymus is susceptible to HIV-1 infection, which compromises thymic function and results in decreased thymic output [3,4]. Also rhesus macaques experimentally infected with SIV show thymic atrophy and reduced thymic output [5-7].

The accessory viral protein Nef plays a key role in efficient viral replication *in vivo* and greatly accelerates disease progression in poorly adapted hosts of primate lentiviruses. Initially, it was shown that an intact *nef* gene is essential for the maintenance of high viral load and disease progression in macaques [8]. Subsequently, defective *nef* genes were detected in several long-term non-progressors of HIV-1 infection with exceedingly low viral loads [9,10]. Moreover, Nef expression alone was sufficient to induce an AIDS-like disease in mice [11-13]. Nef performs multiple activities, such as modulation of cell surface receptors (e.g. CD4, CD8β, CD28, MHC-I and CXCR4), alteration of signal transduction pathways, reducing cellular motility by binding active p21 protein (Cdc42/Rac)-activated kinase (PAK2), and enhancement of viral infectivity and replication [14-18]. HIV-2, SIVsmm, SIVmac and most other SIV Nef proteins are more potent in CXCR4 and CD28 downregulation than HIV-1 Nefs, and in addition capable of downregulating CD3 cell surface expression [19-23]. Previous studies have shown that Nef alleles derived from several HIV-1 group M subtype B strains (e.g. NL4-3, NA7 and LAI) were able to impair T-cell development from CD34^+^ hematopoietic progenitor cells [24-26]. In HIV-1 (LAI) infected bone marrow-liver-thymus humanized (BLT) mice Nef was essential for thymocyte depletion [27]. However, a thorough understanding of the underlying molecular mechanism is lacking and it is unclear whether Nef proteins from other subtypes or groups of HIV-1, HIV-2 and SIV also reduce thymic output.

Therefore, we analyzed a panel of Nef proteins from HIV-1 group M, group O and HIV-2, their simian precursors SIVcpz, SIVgor and SIVsmm, respectively, and SIVmac and SIVblu for their ability to interfere with the development of human thymocytes in fetal thymic organ cultures (FTOC). We show that reduced thymic output is conserved between these HIV and SIV Nef proteins. Domains and residues important for PAK2 binding, such as the C-terminal phenylalanine, were important for reduced thymic output by HIV-1 Nefs that are generally unable to down-modulate CD3 and only weakly affect CXCR4 expression. In comparison, SIVmac Nef mutants that did not bind PAK2 were still able to reduce thymic output because downregulation of CD3 proved to be sufficient for this effect. Finally, mutations in the SIVblu Nef that disrupted both PAK2 interaction and CD3 but not CXCR4 down-modulation did not fully eliminate its effect on thymic output, suggesting that reduced CXCR4 signaling is contributing the effect of SIV Nefs. Our results indicate that reduction of thymic output is a conserved property of primate lentiviral Nef proteins and mediated by effects on multiple cellular factors that are involved in T-cell signaling and migration.

## Results

### Reduction of thymic output is a conserved property of primate lentiviral Nefs

**Figure 1:**
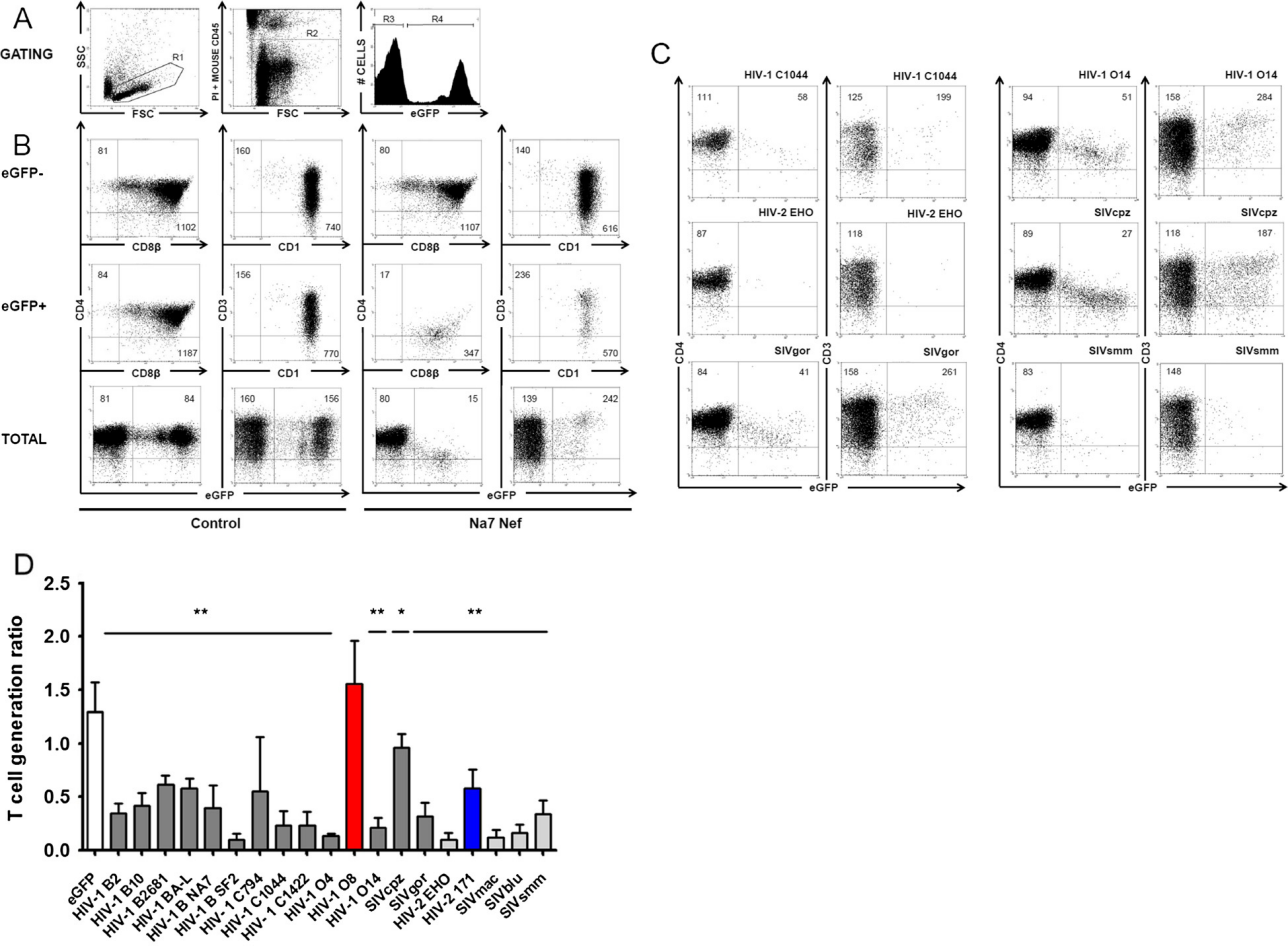
**Reduction of thymic output is a conserved property of primate lentiviral Nef proteins.** Flow cytometric analysis of FTOC initiated with transduced CD34^+^ thymic progenitors. **(A)** Gating strategy: R1 and R2 to gate on living cells of human origin (not staining with anti-mouse CD45 Cy-Chrome). For indicated plots in panel **(B)** eGFP^-^ and eGFP^+^ cells were gated using the histogram gates shown (R3 and R4). **(B)** Dot plots show control eGFP (left) or Nef NA7-IRES-eGFP transduced cultures (right) stained as indicated gated on eGFP^-^ cells (upper plots) and eGFP^+^ cells (middle plots); and eGFP vs. CD4 or CD3 gated on live human cells (lower plots). Quadrants were set to include 99% of non-transduced cells stained with isotypic controls in lower left quadrant. Numbers in plots indicate mean fluorescent intensity (MFI): for upper and middle plots of the marker on the adjacent axis for the total population (all quadrants), for the lower plots MFI’s for the marker on the Y-axis for all eGFP^-^ cells (upper and lower left quadrants) or all eGFP^+^ cells (upper and lower right quadrants) are indicated. **(C)** Representative examples of eGFP vs. CD4 and eGFP vs. CD3 stainings, gated on live human cells of Nef transduced cultures, as indicated. Quadrants and numbers in plots as in lower plots panel B, if sufficient events available. **(D)** T-cell generation ratio (TGR) calculated from Nef transduced cultures, as indicated. Bars represent averages of at least 3 independent experiments, error bars indicate standard deviation. Nef proteins that do not downregulate CD3 are shown in dark grey (in red functional defective mutant HIV-1 O8), those that do in light grey (in blue functional mutant HIV-2 171). Difference compared to control eGFP (white bar) was tested with Kruskal-Wallis with Dunn’s correction for multiple testing, *p < 0.05, **p < 0.005.

We previously reported that expression of Nefs from HIV-1 subtype B strains (NL4.3, NA7 and LAI) in thymic progenitors impaired the development of T cells [24,25]. To assess whether this function is conserved in other primate lentiviral Nef proteins, we analyzed a panel of previously described HIV-1 group M (subtype B and C), HIV-1 group O, HIV-2, SIVcpz, SIVgor, SIVmac, SIVblu and SIVsmm *nef* alleles [28,29] (overview in Table 1). Human thymic CD34^+^ T-cell progenitor cells were transduced with a retroviral vector, co-expressing Nef and the enhanced green fluorescent protein (eGFP) marker from a single bicistronic mRNA, and assayed *in vitro* in FTOC. After 21 days of FTOC culture, CD34^+^ cells transduced with the control vector expressing eGFP, developed into double positive (CD4^+^CD8^+^), CD4^+^CD8^-^ and a few CD4^-^CD8^+^ single positive cells with similar levels of surface marker expression (CD4, CD8β, CD3) compared to that of non-transduced eGFP^-^ cells (Figure 1A, B), as reported before [24,25]. In comparison, HIV-1 Nef expressing cells showed reduced CD4 and CD8β expression. As reported before [24,25], thymocytes were generated in reduced numbers and skewed to higher levels of cell surface CD3 expression compared to eGFP^-^ cells from the same culture (Figure 1B). Since transduced and non-transduced progenitors are not separated before FTOC, the non-transduced thymocytes generated serve as an internal control for the transduced population. Nearly all of the Nef proteins tested reduced thymic output, some to such an extent that hardly any eGFP^+^ and thus Nef expressing cells could be detected (representative dot plots shown in Figure 1C). To measure Nef induced thymic depletion, we calculated the thymocyte generation ratio (TGR) defined as the ratio of the percentage of eGFP^+^ thymocytes harvested to the percentage of eGFP^+^ progenitors put in FTOC (Figure 1D). This TGR parameter was shown before to be a robust measure of T-cell generation potency from transduced progenitors [25]. All HIV-1 group M subtype B and C, as well as two out of three group O Nef proteins showed a pronounced effect on thymopoiesis (Figure 1D). This was due to a developmental defect and not due to toxicity, as Nef expressing thymocytes grown in suspension culture showed comparable survival to control eGFP transduced cells ([24] and data not shown). Remarkably, the natural mutant O8 (HIV-1 group O) Nef protein failed to disturb T-cell development. Of note, this allele is mutated in the valine-glycine-phenylalanine (VGF) domain which is critical for many Nef functions (MHC-I and CXCR4 downregulation, PAK2 binding and cofilin hyperphosphorylation, inhibition of T-cell receptor (TCR) triggering-induced actin ring formation, targeting Lck to the trans-Golgi network and enhancement of infectivity and replication, but not for CD4 downregulation) [29,30]. For HIV-2, EHO Nef reduced T-cell generation (TGR 0.1) much more potently than 171 Nef (TGR 0.6) in comparison to control eGFP transduced cultures (TGR 1.4) (Figure 1D and Table 1). The latter protein was shown to be defective for CXCR4 downregulation, and reduced in its ability to downregulate MHC-I [29] and CD3 (Table 1). All of the SIV Nef proteins tested reduced T-cell generation, albeit with different potency. SIVcpz was the least potent, while SIVmac blocked T-cell development almost completely. To accomplish this, the SIV derived Nefs apparently can interact with the relevant human proteins in the developing thymocytes. Overall, these results demonstrate that the capacity to reduction of thymic output is a conserved feature of highly divergent lentiviral Nef proteins.

**Table 1:** Natural Nef variants used

	**CD3**	**CD4**	**CXCR4**	**MHC-I**	**PAK2**	**TGR**	**References**
**HIV-1**	**-**	**+**	**+/-**	**+**	**+**		
B2	- *	+	+/-	+	ND	0.4 *	[29]
B10	- *	+	+/-	+	ND	0.4 *	[29]
B2681	- *	+	+/-	+	ND	0.6 *	[29]
BA-L	-	+	+/-	+	ND	0.6 *	[29]
NA7	-	+	+/-	+	+/-	0.4	[25,29,31]
SF2	-	+	+/-	+	+	0.1 *	[29,31]
C794	- *	+	+/-	-	ND	0.6 *	[29]
C1044	- *	+	+/-	-	ND	0.2 *	[29]
C1422	- *	+	+/-	+	ND	0.2 *	[29]
O4	- *	+	+/-	+	ND	0.1 *	[29]
O8	- *	+	-	-	ND	1.6 *	[29]
O14	- *	+	+/-	+	ND	0.2 *	[29]
**HIV-2**	**++**	**+**	**+**	**+**			
EHO	++*	+*	+*	+*	ND	0.1 *	[32]
171	+ *	+	-	+/-	ND	0.6 *	[29]
**SIV**		**+**	**++**	**+**			
cpz	-	+	+	+	+	1.0 *	[28,31]
gor	-	+	++	+	ND	0.3 *	[33]
mac	++	+	++	+	+	0.1 *	[28,31,34]
blu	++	+	+	+	+/-	0.2 *	[28,31]
smm	++	+	++	+	+	0.3 *	[28,31,34]

Table shows properties of the Nef proteins (grouped according to origin) used in this study, with reference to published data wherever possible: *indicates this study is the first report. Downregulation of cell surface markers by Nef in transduced PBLs as indicated in column heading is reported semi-quantitatively: from – (no downregulation), over +/- and + to ++ (strong downregulation). PAK2: binding to PAK2 semi-quantitatively: from – (no detectable binding), over +/- to + (strong binding similar to corresponding wild-type). In boldface the expected phenotype for Nef from HIV-1, HIV-2 and SIV origin, when stereotypical. TGR: thymocyte generation ratio, average value is shown, control value is typically about 1.4.

### Disturbance of T-cell development by HIV-1 Nef requires an intact VGF motif and is affected by PAK2 binding

**Figure 2:**
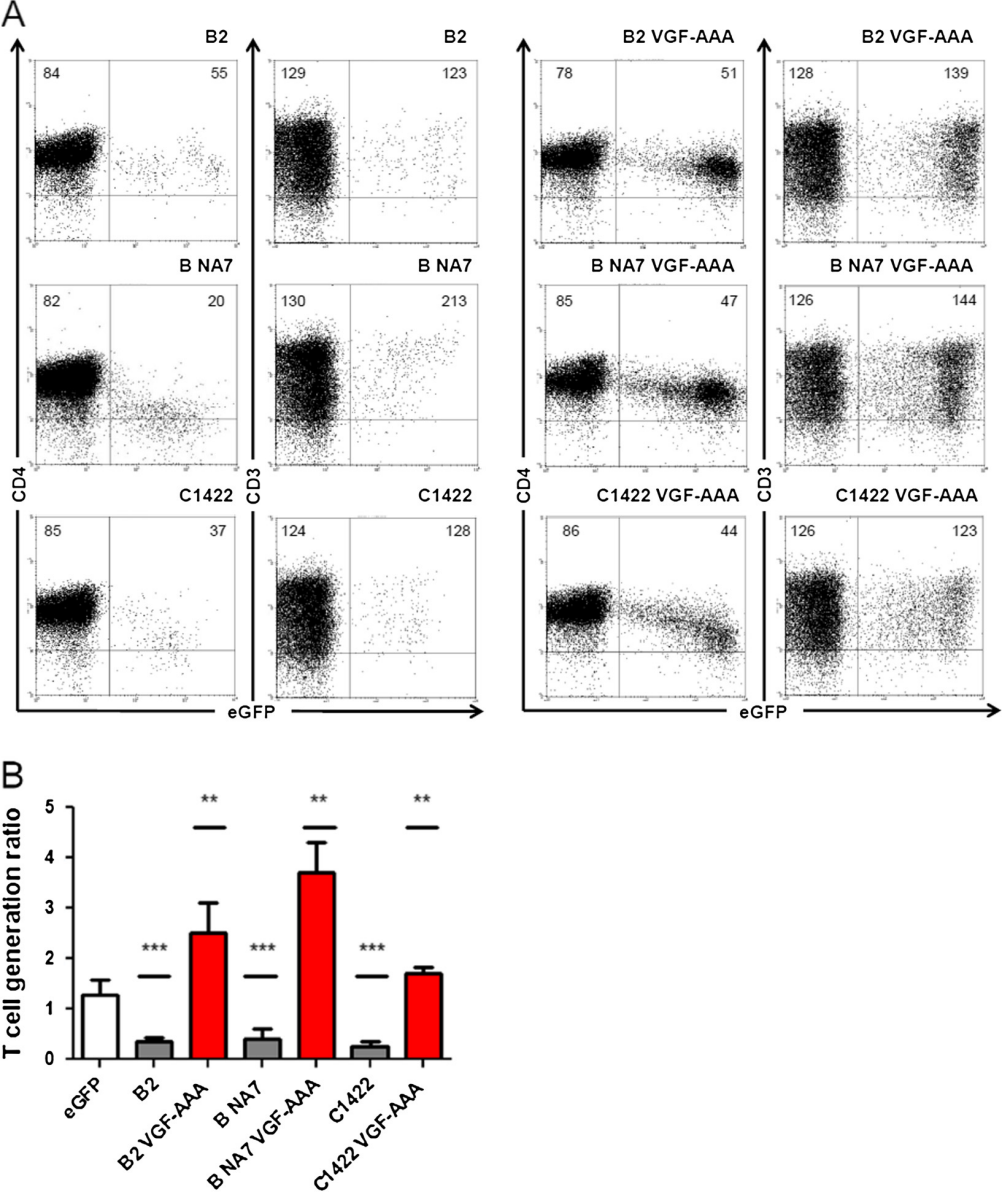
**Reduction of thymic output by HIV-1 Nef requires an intact VGF motif.** Flow cytometric analysis of FTOC initiated with retrovirally transduced CD34^+^ thymic progenitors. **(A)** Dot plots show eGFP vs. CD4 and eGFP vs. CD3 staining gated on live human cells of Nef transduced cultures, as indicated. Quadrants were set to include 99% of non-transduced cells stained with isotypic controls in lower left quadrant. Numbers in plots indicate mean fluorescent intensity for the marker on the Y-axis (CD4 or CD3) for all eGFP^-^ cells (upper and lower left quadrants) or all eGFP^+^ cells (upper and lower right quadrants) are indicated. **(B)** T-cell generation ratio calculated from Nef transduced cultures, as indicated. Bars represent averages of at least 3 independent experiments, error bars indicate standard deviation. Wild-type Nef proteins are shown in dark grey, VGF-AAA mutants in red. Difference compared to control eGFP (white bar) was tested with Mann–Whitney U test, **p < 0.005, ***p < 0.0005.

The Nef protein of the O8 strain is deleted in the VGF domain and in the first proline of the neighboring polyproline domain (PxxP). Although the importance of the latter domain for deregulation of T-cell development was shown before [25], the functional relevance of the VGF motif in this respect is unknown. To investigate the relevance of this VGF domain for reduced thymic output by Nef alleles from various group M HIV-1 strains, we mutated the VGF motif to an alanine triplet (VGF-AAA) in subtype B (NA7 and B2), and subtype C (C1422) Nefs as described before [29]. Table 2 shows an overview of all mutants used in this study, their ability to downregulate the cell surface markers assessed in primary CD4+ T cells (PBLs iso primary CD4+ T cells), to bind PAK2 and their effect on thymic output as measured by TGRs. When the VGF mutants were expressed in thymocytes, cell surface CD4 and CD3 levels were similar to that of thymocytes expressing wild-type Nefs (Figure 2A). However, the number of thymocytes generated was significantly increased (Figure 2B). This is in line with our previous observations, indicating that domains important in the signaling properties of Nef, such as binding to SH3 domains or PAK2, are of importance for reduced thymic output in the presence of Nef [25].

**Table 2:** Artificial Nef mutants used

	**CD3**	**CD4**	**CXCR4**	**MHC-I**	**PAK2**	**TGR**	**Reference**
**HIV-1**	-	+	+/-	+			
B2 VGF-AAA	- *	+ *	- *	- *	ND	2.5 *	This study
NA7 VGF-AAA	- *	+	-	-	-	3.7 *	[29]
C1422 VGF-AAA	- *	+ *	- *	- *	ND	1.7 *	This study
NA7 F191H	-	+	- *	+	+/-	1.0 *	[35]
NA7 F191R	-	+	- *	+	-	1.7 *	[35]
SF2 F195I	- *	+	- *	+	+/-	0.3 *	[36]
SF2 F195A	- *	+	- *	+	-	0.7 *	[37]
**SIV**		**+**	**++**	**+**			
mac AXXP	++ *	+	+/- *	+	+ *	0.3 *	[38]
mac AXXA	++	+	- *	+	+/- *	0.2 *	[38,39]
mac PXXA	++ *	+	- *	+	+/- *	0.2 *	[38]
mac H121R	++ *	+ *	- *	- *	+/- *	0.3 *	This study
mac Y221R	++ *	+ *	- *	- *	+/- *	0.6 *	This study
mac H121R/Y221R	++ *	+ *	- *	- *	- *	0.5 *	This study
mac ∆153	++	-	- *	-	- *	0.2 *	[39]
mac ∆183	-	-	- *	-	- *	1.2 *	[39]
blu RR-AA	-	+ *	+ *	+/-*	- *	0.5 *	[40]

Table shows properties of the mutated Nef proteins (grouped according to origin) used in this study, with reference to published data wherever possible: *indicates this study is the first report. Downregulation of cell surface markers by Nef in transduced CD4^+^ PBLs as indicated in column heading is reported semi-quantitatively: from – (no downregulation), over +/- and + to ++ (strong downregulation). PAK2: binding to PAK2 semi-quantitatively: from – (no detectable binding), over +/- to + (strong binding similar to corresponding wild-type). In boldface the expected phenotype for Nef from HIV-1, HIV-2 and SIV origin, when stereotypical. TGR: thymocyte generation ratio, control value is typically about 1.4. ND: not determined.

**Figure 3:**
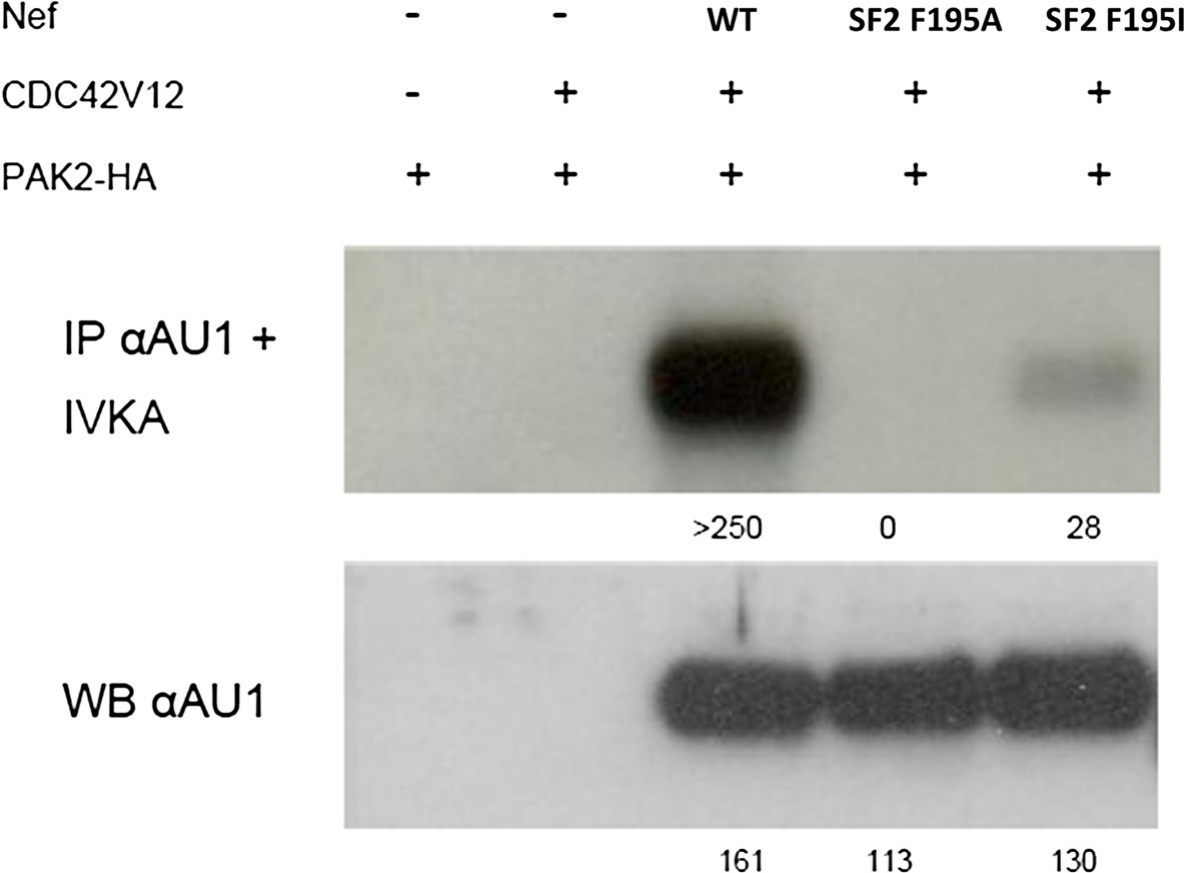
**SF2 Nef proteins mutated in F195 show reduced PAK2 binding.** In vitro kinase assay of AU1-tag immunoprecipitated Nef from cells transfected with SF2 Nef, constitutive active CDC42, and HA-tagged PAK2, as indicated. Upper panel shows Nef co-immunoprecipitated PAK2 activity, lower panel anti-AU1 staining of cell lysates on Western blot (numbers indicate densitometric quantification).

**Figure 4:**
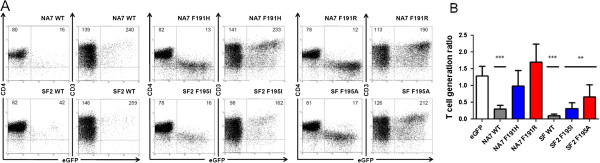
**Reduction of thymic output by HIV-1 Nef is affected by PAK2 binding.** Flow cytometric analysis of FTOC initiated with retrovirally transduced CD34^+^ thymic progenitors. **(A)** Dot plots show eGFP vs. CD4 and eGFP vs. CD3 staining gated on live human cells of Nef transduced cultures, as indicated. Quadrants were set to include 99% of cells stained with isotypic controls and eGFP cells in lower left quadrant. Numbers in plots indicate mean fluorescent intensity for the marker on the Y-axis (CD4 or CD3) for all eGFP^-^ cells (upper and lower left quadrants) or all eGFP^+^ cells (upper and lower right quadrants) are indicated. **(B)** T-cell generation ratio calculated from Nef transduced cultures, as indicated. Bars represent averages of at least 3 independent experiments, error bars indicate standard deviation. Wild-type Nef proteins are shown in dark grey, mutants showing reduced PAK2 binding in blue, mutants showing no detectable PAK2 binding in red. Difference compared to control eGFP (white bar) was tested with Mann–Whitney U test, **p < 0.005, ***p < 0.0005.

To investigate this further, we used HIV-1 subtype B Nef single point mutants known to be important for PAK2 binding while not affecting other known Nef activities. These proteins are mutated in the most C-terminal phenylalanine residues F191 and F195 for HIV-1 NA7 and SF2 strains respectively, which are important residues of the HIV-1 Nef PAK2 activating structural motif (PASM) [20,36,41]. As described before [35], the NA7 F191H mutant has a reduced affinity for PAK2 binding, while the NA7 F191R mutant does not bind PAK2 at detectable levels. Similarly, F195 in SF2 Nef was described to be important for PAK2 binding [20,36,37,42]. To demonstrate AU1-tagged SF2 Nef proteins (wild-type, F195I and F195A) differed in binding capacity for PAK2, they were transfected in 293 T cells to measure activated PAK2 binding as described before [43,44]. While in this setting residual binding of PAK2 to SF2 Nef F195I was detected, binding to SF2 Nef F195A was undetectable (Figure 3). When assayed for their effect on T-cell development, reduced PAK2 binding did not affect the CD3 or CD4 cell surface staining profiles (Figure 4A) but did diminish the effect on T-cell development (Figure 4B) in comparison to wild-type Nef. However, while NA7 F191R did not affect T-cell generation at all, SF2 F195A was still partially active (TGR 0.7) despite the fact that no detectable PAK2 activity was bound (Figure 3). This suggests that in the case of SF2 either a minute but undetectable PAK2 activity bound to Nef is sufficient to affect T-cell development, or that other Nef functions contribute to reduced thymic output.

### Mutants SIVmac Nefs lacking PAK2 interaction still hamper T-cell development

**Figure 5:**
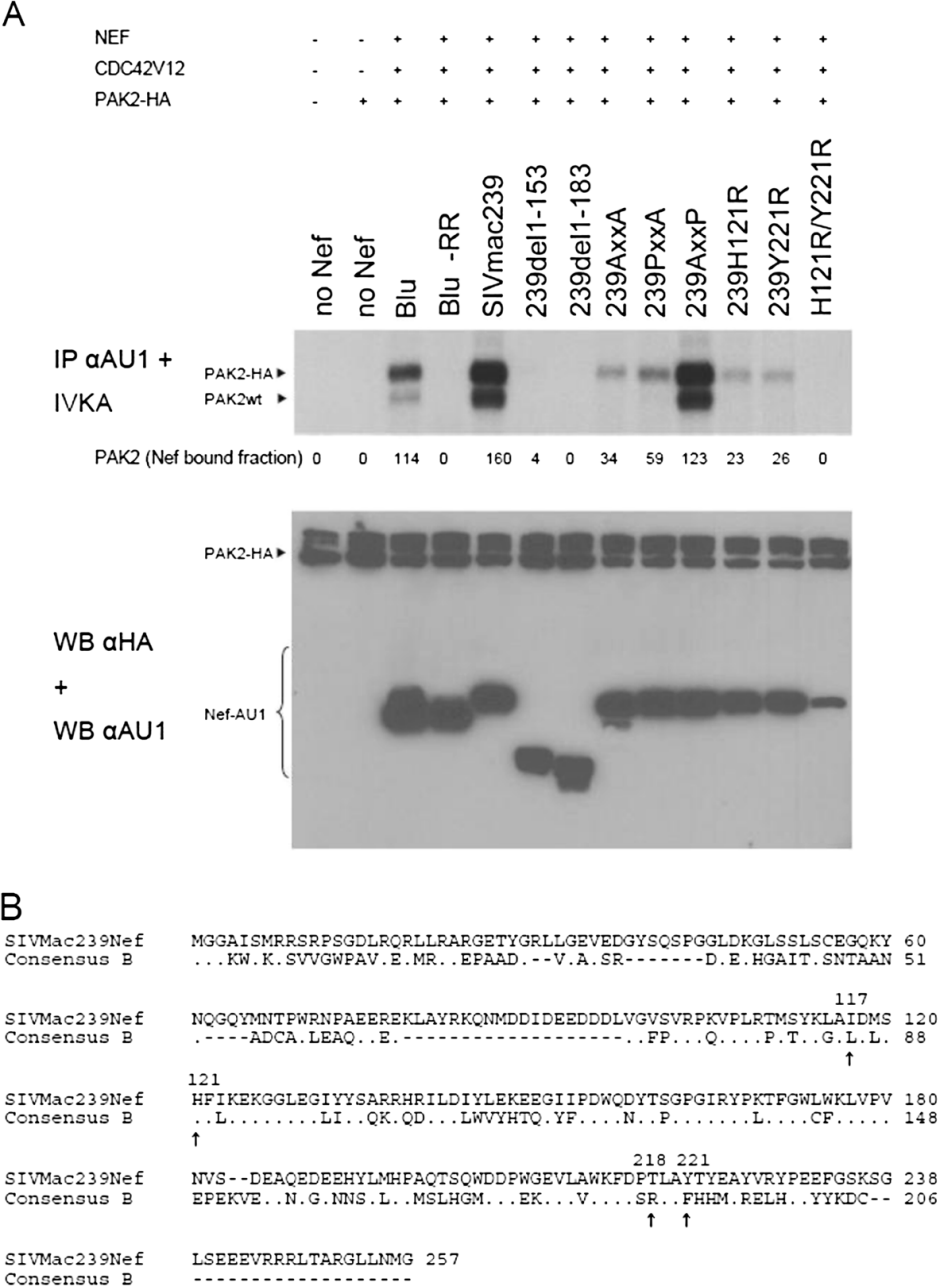
**PAK2 binding of mutant Nef proteins and positional homologs of PAK2 activating structural motif. (A)** In vitro kinase assay of AU1-tag immunoprecipitated Nef from cells transfected with Nef, constitutive active CDC42, and HA-tagged PAK2, as indicated. Upper panel shows Nef co-immunoprecipitated PAK2 activity (figures indicate densitometric quantification), lower panel anti-HA and anti-AU1 staining of cell lysates on Western blot. **(B)** Alignment of SIVmac239 Nef to a consensus HIV-1 subtype B Nef sequence derived from a database of 1643 sequences previously described [36]. Arrows denote SIVmac239 Nef amino acid residues 117, 121, 218 and 221 which are positional homologs to HIV-1 subtype B Nef 85, 89, 188 and 191.

**Figure 6:**
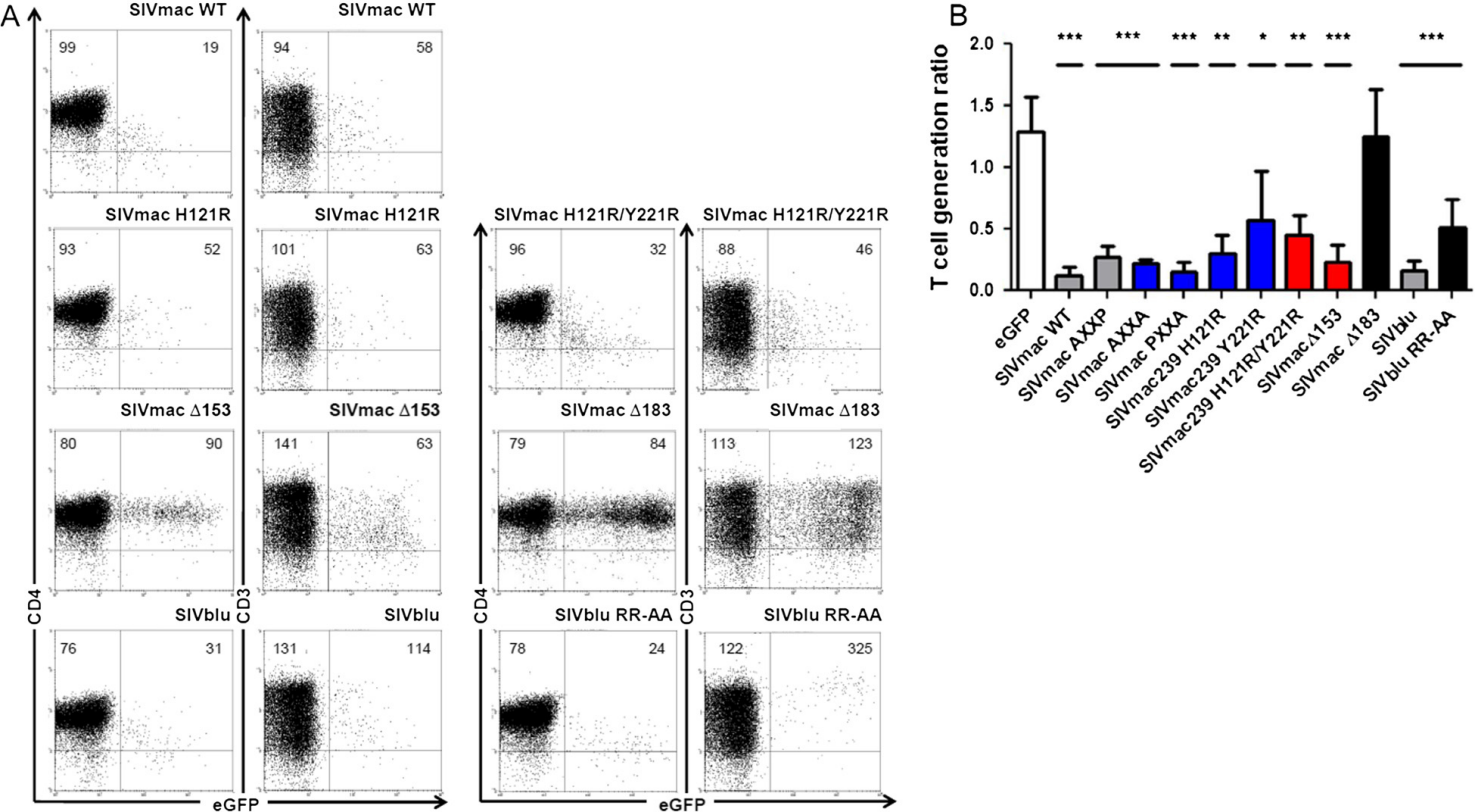
**PAK2 binding is not essential and downregulation of CD3 is sufficient to hamper T-cell development by SIV Nef.** Flow cytometric analysis of FTOC initiated with retrovirally transduced CD34^+^ thymic progenitors. **(A)** Dot plots show eGFP vs. CD4 and eGFP vs. CD3 staining gated on live human cells of Nef transduced cultures, as indicated. Quadrants were set to include 99% of non-transduced cells stained with isotypic controls in lower left quadrant. Numbers in plots indicate mean fluorescent intensity for the marker on the Y-axis (CD4 or CD3) for all eGFP^-^ cells (upper and lower left quadrants) or all eGFP^+^ cells (upper and lower right quadrants) are indicated. **(B)** T-cell generation ratio calculated from Nef transduced cultures, as indicated. Bars represent averages of at least 3 independent experiments, error bars indicate standard deviation. Nef proteins that bind PAK2 and downregulate CD3 are shown in dark grey, mutants showing reduced PAK2 binding in blue, mutants with no detectable PAK2 binding in red, mutants showing no detectable PAK2 binding nor CD3 downregulation in black. Difference compared to control eGFP (white bar) was tested with Mann–Whitney U test, *p < 0.05, **p < 0.005, ***p < 0.0005.

Since PAK2 interaction with HIV-1 Nefs seems to play an important role in reduced thymic output, we investigated whether this mechanism is conserved in other lentiviral Nef proteins. First, we analyzed the effect of mutations in prolines P104 and P107 of the polyproline stretch (PxxP) of SIVmac239 Nef. The mutation of P104 was described to marginally affect PAK2 binding while P107 was shown to be essential for PAK2 binding [45]. Surprisingly, we found that P107A and the P104A/P107A double mutant (PxxA and AxxA) could still bind PAK2 albeit with strongly reduced efficacy (Figure 5A). We therefore sought other mutants that were selectively defective for PAK2 binding. As mentioned above, we previously described a PASM in HIV-1 Nef comprised of amino acids L85, H89, R188 and F191 [36,46]. When we aligned SIVmac239 protein sequence with a consensus subtype B Nef sequence [36], SIVmac239 Nef residues I117, H121, T218 and Y221 were revealed as potential candidates involved in the binding of PAK2 due to their positional homology to PASM residues of HIV Nef (Figure 5B). To determine whether the four candidate SIV Nef amino acids are indeed involved in PAK2 binding, we mutated each one to three different substitutions and measured PAK2 binding. While substitution at all positions could reduce PAK2 binding (Additional file 1: Figure S1), our results showed that the H121R and Y221R mutant proteins expressed well and were strongly reduced in PAK2 binding. These identified residues therefore constitute the PASM of SIVmac, homologous to that of HIV-1 Nef. We also generated a double mutant that was not able to bind active PAK2 at all (Figure 5A). The H121R, Y221R single and double mutants were capable of downregulating the expression of cell surface CD4, CD3, CD28 and to a lesser extent MHC-I to similar levels as SIVmac239 Nef in cell lines and infected human peripheral mononuclear cells (Additional file 1: Figure S2) and thymocytes (Figure 6A). In addition, the mutations of H121R/Y221R did not affect downregulation of MCH class II and upregulation of invariant chain Ii (Additional file 1: Figure S2D), nor hyperactivation of NFAT (data not shown) by Nef. By contrast, downregulation of CXCR4 was compromised (Table 2).

We found that individual or combined mutations in the PxxP motif or in residues H121 and Y221 of the SIVmac239 disrupted PAK2 interaction, but had little if any effect on reduction of thymic output (Figure 6B). This shows that PAK2 binding is not essential, or that residual but undetectable PAK2 binding is sufficient, for the reduction of thymic output by SIVmac239 Nef. In contrast to HIV-1 Nef, SIVmac Nef downregulates CD3 in CD4^+^ lymphocytes [28]. Since CD3 is important for TCR signal transduction and T-cell development critically depends on TCR signaling [47], we hypothesized that this function may explain why loss of PAK2 binding did impair the effect of SIVmac239 Nef on thymic output. To examine this we utilized two previously described SIVmac Nef deletion mutants: *nef*_∆153_ and *nef*_∆183_[48]. The Nef_∆153_ protein does not downregulate CD4, CD28 and MHC-I or enhance viral infectivity and replication but still reduces CD3 surface expression. By contrast, the *nef*_∆183_ allele has also lost the latter function due to an additional deletion of 10 amino acids. These phenotypical characteristics were confirmed in transduced thymocytes that developed in FTOC (Figure 6A) and in CD4^+^ PBLs (Table 2). As expected, both proteins were unable to bind PAK2 (Figure 5). However, Nef_∆153_ still reduced thymic output, whereas Nef_∆183_ was inactive (Figure 6B). Thus CD3 downregulation by SIVmac Nef is sufficient to block T-cell development. The experiments reported above cannot exclude other SIVmac239 Nef functions to contribute to this effect. However, no SIVmac mutant Nef protein was available that selectively lost PAK2 and CD3 downregulating properties.

Finally, we also tested the Nef allele of SIVblu infecting blue monkeys (*Cercopithecus mitis*) and a mutant thereof (RR-AA) containing amino acid changes R129A and R130A. In comparison to the wild-type protein, the SIVblu RR-AA mutant lost its ability to downregulate CD3 in thymocytes and PBLs (Figure 6A and Table 2) and bind PAK2 (Figure 5A). Nonetheless, the protein still impaired T-cell generation, albeit to a lower extent when compared to SIVblu wild-type Nef (TGR 0.5 and 0.2 respectively, Figure 6B). As CXCR4 signaling is important for T-cell development [49,50], the downregulation of CXCR4, conserved in SIVblu RR-AA possibly contributes to this residual effect.

## Discussion

We show here that expression of highly diverse primate lentiviral Nef proteins in human T-cell progenitors reduces their development into T cells. Primate lentiviral Nefs may impair thymic output by at least three mechanisms including PAK2 interaction and downregulation of CD3 or CXCR4. The effect of HIV-1 Nefs on T-cell development is mainly dependent on PAK2 interaction since they generally lack the CD3 down-modulation function and have only weak effects on CXCR4. In contrast, SIV Nefs lacking PAK2 interaction maintain the ability to reduce thymic output, either by downregulation of CD3 or CXCR4 .

Reduced thymic output most likely contributes to the failure to compensate for increased destruction of CD4^+^ T cells in lymphoid tissues in HIV-1 infected patients, ultimately resulting in AIDS [51]. Thymic progenitors have been shown to be infected at significant rates in patients [3,4,52]. In these cells, expression of Nef may block the generation of differentiated T cells. Also in HIV-2 infected patients, reduction in recent thymic emigrants was seen in younger patients (<45 years of age), as measured by signal joint/beta T-cell receptor excision circle levels in circulating lymphocytes. T-cell development is process requiring differentiation of CD34+ thymic precursor cells into successive stage of thymocytes undergoing selection to express suitable TCRs [47,53]. In this process, interaction of developing thymocytes with the environment through cell surface receptors while migrating through the organ is of pivotal importance. It is therefore conceivable that a viral protein that affects migration (through PAK2 binding) and expression of receptors important for T-cell development (CD3 and CXCR4) would be capable of disrupting T-cell generation. Remarkably, all SIV Nef proteins tested in this study, including those from SIV that do not provoke disease in their natural host, were able to hamper human T-cell development, indicating that required interactions with host proteins are conserved across species. While downregulation of CD3 will impair thymic output, reduced immune activation in the periphery might outweigh this so that SIV infection by CD3 downregulating strains is marked by a relative preservation of peripheral CD4^+^ T-cell counts in the natural hosts. Primate lentiviruses, however, differ in Nef function. While most Nef proteins downregulate CD4 and MHC-I, and bind to kinases such as PAK2, HIV-1 and its simian precursor SIVcpz are peculiar in that the encoded Nef proteins have lost the ability to downregulate CD3 [16]. This is in contrast to Nefs encoded by HIV-2 and its closest simian counterpart SIVsmm/SIVmac. In addition, the latter also downregulate CXCR4 more potently than HIV-1/SIVcpz Nef [34]. Our previous work with HIV-1 subtype B Nef showed that downregulation of CD4, CD8β or MHC-I did not correlate with the reduced thymic output by Nef [25]. However, we showed several motifs involved in PAK2 binding (acidic cluster, polyproline domain, R106) to be important for deregulating T-cell development. We recently identified a VGF domain, linking the acidic cluster and the polyproline motif, important for many Nef functions including PAK2 binding [29]. Here we show that this VGF motif is also essential for the effect of HIV-1 Nef on thymic output. To investigate the role of PAK2 more specifically, we used point mutants of the C-terminal phenylalanine known to affect PAK2-Nef binding, i.e. F191 (NA7)/F195 (SF2). The NA7 F191R mutant Nef lost its effect on thymic output completely, demonstrating that at least in some HIV-1 Nef alleles, PAK2 binding is crucial for reducing thymic output. However, F195A mutant of SF2 Nef maintained some activity in reducing thymic output (TGR 0.7 compared to TRG 0.1 for wild-type SF2), despite the complete loss of detectable PAK2 binding.

SIVmac Nefs, which in contrast to HIV-1 and SIVcpz Nef, downregulate CD3 and more potently downregulate CXCR4, also bind PAK2 and reduced thymic output very efficiently (TGR 0.3 or below). To investigate the importance of PAK2 binding by these proteins we used previously described and newly developed mutants. The SIVmac Nef double mutant P104A/P107A still bound detectable PAK2 activity, which could explain the observed effect on T-cell development (TGR 0.2). To rule this out, we developed mutant proteins based on the positional homology with the HIV-1 PAK2 activating structural motif (PASM) we described before [36]. In SIVmac Nef, 4 residues were identified that critically contribute to PAK2 binding. Similar to HIV-1 Nef, SIVmac thus forms a PASM that points to the relevance of this binding for Nef function [31], and which allowed us to create selective mutants. Nef-PAK2 binding was strongly reduced in the H121R and Y221R single mutants, and undetectable for the double mutant. This double mutant still significantly reduced thymic output (TGR 0.5) compared to eGFP expressing control cultures (TGR 1.4), albeit with reduced potency when compared to wild-type SIVmac (TGR 0.1). Thus, different from what we observed with HIV-1 NA7 Nef, SIVmac239 Nef proteins mutated in residues relevant for PAK2 binding (P104, P107, H121 and Y221) still reduce thymic output, albeit at slightly reduced levels (TGR from 0.2 to 0.6) compared to wild-type SIVmac239 Nef (TGR 0.1). Compared to wild-type, these PAK2-binding Nef mutants were attenuated in downregulation of CXCR4 but fully active as wild-type to downregulate CD3. Here we demonstrate that an internally deleted SIVmac Nef protein that lost all of its known functions except downregulation of CD3 from the cell surface (*nef*_∆153_) [48] can block T-cell development (TGR 0.1). Downregulation by SIVmac239 Nef of CD3 is thus sufficient to reduce thymic output, and might compensate for defects in PAK2 binding and CXCR4 downregulation in the mutants discussed above.

Downregulation of CXCR4 will most likely contribute to impaired T-cell development, given the essential role of SDF1α/CXCR4 signaling in this process [49,50]. This is underscored by the residual effect on thymic output of the SIVblu RR-AA Nef mutant, that lost the capacity to bind PAK2 and downregulate CD3 but retained CXCR4 downregulation, compared to the wild-type protein. Possibly, the effects of Nef on thymic output might also involve the re-localization of the tyrosine kinase Lck away from plasma membrane to the trans-golgi network [54]. Since Nef-mediated retargeting of Lck alters its signal transduction properties [55,56] and association of this tyrosine kinase to the CD4/CD8 co-receptor is important for thymocyte selection [57], Nef may disrupt signaling events essential for thymocyte generation via this mechanism. In support of such a scenario, at least in transgenic mice, overexpression of constitutive active Lck abrogated the effects of Nef on T-cell development [58]. Testing the relative contribution of CXCR4 downregulation or of Lck re-localization to the reduction of thymic output by Nef will require the identification of mutants or variants of the viral protein that are selectively defective for these activities.

## Conclusion

In conclusion, we showed that reduced thymic output is a conserved feature of primate lentiviral Nef proteins. For HIV-1 Nef, active PAK2 binding is crucial to this effect, while the CD3 downregulation e.g. by HIV-2 Nef and other SIV Nef proteins is sufficient to provoke a block in T-cell development. The latter proteins will in addition downregulate CXCR4, what might contribute to their effect on T-cell development. Thus depending on the context, multiple Nef functions contribute to the reduction of thymic output, what appears to be a well conserved and likely relevant effect of primate lentivirus Nef proteins. Infection of the thymus, leading to expression of Nef in developing thymocytes, may contribute to this decline. Further mechanistic exploration of Nef function in developing and differentiated T cells is needed to elucidate the importance of Nef-mediated reduced thymic output for the pathogenesis of AIDS.

## Methods

### Viruses, vectors and molecular clones

HIV-1 group M and group O isolates were obtained from the AIDS Research and Reference Reagent Program, while the HIV-2 strains were previously isolated from patients attending the AIDS clinic at the Institute of Tropical Medicine in Antwerp, Belgium, with the approval of the ethical committee after written informed consent. The *nef* genes from these isolates were available in the LZRS-IRES-eGFP retroviral vector constructed before [29]. Similarly, *nef* sequences from SIVcpz (GAB2), SIVmac (239), SIVsmm, SIVgor, SIVblu and derived mutants [33,39,40] were amplified and cloned into the same vector. In some cases tagged proteins and new mutants were created by overlap extension PCR or site-directed mutagenesis (primers in Additional file 1: Table S1). Double mutant SIVmac 239 H121R/Y221R was constructed starting from cloned single mutant PCR amplicons, taking advantage of the PsiI restriction site that is internal relative to the 121 and 221 residues. Single and double mutant *nef* were further subcloned by introducing the BglII/NdeI SIV *nef* mutated fragments into a construct comprised of a ClaI/EcoRI fragment from SIVmac239 FL SPX (kindly provided by Dr. R. Desrosiers, Harvard Medical School, Boston). These were used to clone by PCR the mutant *nef* sequences in expression vectors and retroviral vector plasmids, and in replication competent HIV-1 reporter virus (see Additional file 1: Methods). For in vitro kinase assays (IVKA), *nef* sequences were amplified with primers containing an AU1 tag sequence for C-terminal tagging and cloned in the pCG expression plasmid as described before [39]. The integrity of the constructs and the *nef* genes was confirmed by Sanger sequencing, protein expression was evident from Western blot and/or biological activity.

### Retroviral gene transfer

The Phoenix-Amphotropic packaging cell line was transfected with LZRS retroviral vector plasmids to produce *nef*-IRES-*eGFP* bicistronic mRNA encoding retroviral vectors as previously described [24]. Isolation, culture and transduction of CD34^+^ thymus cells was performed as described before [24,25]. Briefly, CD34^+^ cells were seeded on RetroNectin (Takara Biomedicals, Otsu Shiga, Japan) coated culture plates with half of the medium volume replaced by retroviral supernatants, supplemented with SCF (10 ng/mL) and IL-7 (10 ng/mL). Transduced CD34^+^ cells were used after 24 hours for fetal thymus organ cultures (FTOC). The excess of transduced progenitor cells, which were not used in FTOC, were kept in culture for 72 hours, to determine the transduction efficiency that varied between 10% and 20%. Child thymus tissue, removed during cardiac surgery, was obtained and used following the guidelines of the Medical Ethical Commission of Ghent University Hospital. Written informed consent was provided according to the Declaration of Helsinki.

### Fetal thymic organ cultures (FTOC) and flow cytometry

Isolation of thymic lobes from fetal nonobese diabetic (NOD)-SCID mice and subsequent murine FTOC were performed and analyzed as described previously [25,59]. Mice were treated and used in agreement with the guidelines of the local ethical committee. To asses thymic depletion due to Nef expression we calculated the thymocyte generation ratio (TGR), which is defined by the ratio of the percentage of eGFP^+^ thymocytes harvested to the percentage of CD34^+^ eGFP^+^ progenitors that were put in FTOC [25,59]. To asses cell surface modulation by the different Nef proteins, we isolated CD4^+^ cells (PBLs) from buffy coat peripheral blood mononuclear cells (normal blood donors, Red Cross, Ghent, Belgium) by negative selection using paramagnetic beads (MACS; Miltenyi Biotec, Bergish Gladbach, Germany). After isolation, the cells were cultured for 3 days in RPMI medium supplemented with 2 mM L-glutamin, 10% heat-inactivated fetal calf serum, phytohemagglutinin (1 μg/mL; Thermo Fisher Scientific, Waltham, USA), 20 ng/mL IL-2 (Peprotech, Rocky Hill, USA), 100 U/mL penicillin, and 100 g/mL streptomycin. Thereafter, PBLs were transduced with retroviral vectors on Retronectin RetroNectin (Takara Biomedicals, Otsu Shiga, Japan) coated culture plates with half of the medium volume replaced by retroviral supernatants, supplemented with IL-2 to keep final cytokine concentrations constant. After another 2 days of culture, cells were harvested and stained for flow cytometry. Antibodies used were directly labeled mouse monoclonals anti-CD1 phycoerythrin [PE] and anti-HLA-A, -B,- C-PE (Becton Dickinson, Erembodegem, Belgium), anti-CD8β-PE (Coulter, Miami, FL) anti-CD3 allophycocyanin [APC], anti-CD4-APC and anti-CXCR4-PE-Cy7 (Miltenyi Biotec, Bergisch Gladbach, Germany), flow cytometers used were FACSCalibur (Becton Dickinson) and MACSQuant (Miltenyi Biotec).

### Western blotting and in vitro kinase assay (IVKA)

Western blot and IVKA was performed as described before [43]. Briefly, HA-tagged PAK2, dominant-active Cdc42V12 and AU1-tagged Nef were transfected into 293 T cells. Lysates of transfected cells were in part subjected to immunoblot to quantify total amount of tagged proteins, and in part used for anti-AU1 Babco, Richmond, CA) immunoprecipitation using protein G-Sepharose beads. Immunoprecipitate was incubated to detect autophosphorylation activity of PAK2 in the presence of ^32^P-ATP by autoradiography of SDS-PAGE.

### Statistics

Analyses were performed using the GraphPad Prism version 5.00 statistical software (GraphPad Software Inc., La Jolla, CA, USA), non-parametric tests used were Mann–Whitney U test and Kruskal-Wallis with Dunn’s correction for multiple testing.

## Abbreviations

BLT: Bone marrow-liver-thymus; EFGP: Enhanced green fluorescent protein; FTOC: Fetal thymic organ culture; HIV: Human immunodeficiency virus; IVKA: In vitro kinase assay; MFI: Mean fluorescence intensity; NOD: Nonobese diabetic; PAK2: p21 protein (Cdc42/Rac)-activated kinase; PASM: PAK2 activating structural motif; PBLs: Primary blood CD4+ T lymphocytes; SIV: Simian immunodeficiency virus; TCR: T-cell receptor; TGR: Thymocyte generation ratio.

## Competing interests

The authors declare that they have no competing interests.

## Authors’ contributions

AVN performed cell surface marker flow cytometry, assisted to FTOC, analyzed data and helped to draft the manuscript, KKA constructed and sequenced vectors, assisted to FTOC and analyzed data, VS constructed and sequenced vectors, assisted to FTOC and analyzed data, MS constructed and sequenced vectors, performed cell surface marker flow cytometry and NFAT assays, and analyzed data, EO constructed and sequenced vectors, performed Western blots and IVKA, performed sequence alignment, conceived PASM studies and analysed data, JS constructed and sequenced vectors, IVDWB assisted to FTOC and analysed data, EN performed FTOC and produced vector stocks, HV produced vector stocks and sequenced constructs, KVL sequenced constructs, TT analysed data, KP performed Western blots and IVKA, KS analyzed data, JVG conceived PASM studies and analyzed data, OTF analyzed data, FK conceived derivation of functional SIV Nef mutants, analyzed data and helped to draft the manuscript, BV designed of the study, analyzed data, performed statistical analysis and wrote the manuscript. All authors read and approved the final manuscript.

## Supplementary Material

Additional file 1 **Supplementary Methods. Table S1.** Primers used to generate mutant and tagged Nef protein expression constructs. **Figure S1.** SIVmac239 Nef displays a PAK2-activating structural domain surface and **Figure S2.** Cell surface marker modulation by SIVmac239 mutated in PAK2-59 activating structural domain surface. Click here for file
