# Supplementary material for: Primate lentiviral Nef proteins deregulate T-cell development by multiple mechanisms
Source: Retrovirology. 2013 Nov 15;10:137. doi: 10.1186/1742-4690-10-137 (PMC3906981; doi:10.1186/1742-4690-10-137)
Supplement: Additional file 1 — Supplementary Methods. Table S1. Primers used to generate mutant and tagged Nef protein expression constructs. Figure S1. SIVmac239 Nef displays a PAK2-activating structural domain surface and Figure S2. Cell surface marker modulation by SIVmac239 mutated in PAK2-59 activating structural domain surface. [file 1742-4690-10-137-S1.pdf]

Additional files

Supplementary Methods

PAK2 binding by SIVmac239 mutated in residues of the putative PAK2 activating structural motif

SIVmac239 Nef residues I117, H121, T218 and Y221 were each mutated to three different substitutions by overlapping PCR. The resulting constructs were cloned into pCDNA3.1 and transfected into 293T cells as previously described [1]. Western blot of lysates was revealed with anti-SIVmac Nef rabbit antiserum [2] followed by horseradish peroxidase (HRP)-conjugated anti-rabbit antibodies. HRP conjugates were visualized by using enhanced chemiluminescence. In vitro kinase assay was essentially performed as previously described [1]. Immunoprecipitations were performed using the rabbit anti-SIVmac Nef antiserum. Nef/PAK-2 complexes were immunoadsorbed and assayed for autophosphorylation activity. Phosphorylated proteins were resolved by sodium dodecyl sulfate-polyacrylamide gel electrophoresis, and dried gels were then exposed to a phosphorimager screen.

Assessment of cell surface marker modulation by SIVmac239 Nef mutated in H121R and/or Y221R Nef

Cloned single and double mutant SIVmac 239 H121R/Y221R *nef* sequences were cloned into the bicistronic cytomegalovirus-based pCG expression vector coexpressing the GFP and Nef and in replication competent HIV-1 NL4-3 based proviral contructs in

the Nef reading frame [3, 4]. pCG plasmids were used to transfect cell lines (Jurkat and THP-I) [5]. HIV/SIV Nef/eGFP viral stocks were generated by transfection in 293T cells and used to infect stimulated normal donor peripheral blood mononuclear cells (PBMC) as described before [4]. After cell culture, transfected or infected cells were stained and analysed by flow cytometry as described before [3].

Supplementary references

1. O'Neill E, Kuo LS, Krisko JF, Tomchick DR, Garcia JV, Foster JL: **Dynamic evolution of the human immunodeficiency virus type 1 pathogenic factor, Nef.** *J Virol* 2006, **80:**1311-1320.
2. Garcia JV, Foster JL: **Structural and functional correlates between HIV-1 and SIV Nef isolates.** *Virol* 1996, **226:**161-166.
3. Schindler M, Munch J, Brenner M, Stahl-Hennig C, Skowronski J, Kirchhoff F: **Comprehensive analysis of Nef functions selected in simian immunodeficiency virus-infected macaques.** *J Virol* 2004, **78:**10588-10597.
4. Schindler M, Munch J, Kutsch O, Li H, Santiago ML, Bibollet-Ruche F, Muller-Trutwin MC, Novembre FJ, Peeters M, Courgnaud V, et al: **Nef-mediated suppression of T cell activation was lost in a lentiviral lineage that gave rise to HIV-1.** *Cell* 2006, **125:**1055-1067.
5. Schindler M, Wildum S, Casartelli N, Doria M, Kirchhoff F: **Nef alleles from children with non-progressive HIV-1 infection modulate MHC-II expression more efficiently than those from rapid progressors.** *AIDS* 2007, **21:**1103-1107.

Supplementary Table 1: primers used to generate mutant and tagged Nef protein expression constructs

	**Forward**	**Reverse**
**SIVmac239 H121R**	5’-ccgtctggagatctgcgacagagac-3’	5’-cttttataaatctagacatgtctattgccaatttg-3’
**SIVmac239 Y221R**	5’-ccgtctggagatctgcgacagagac-3’	5’-catatgcctcataagtcctggccagagttgg-3’
**HIV1 SF2-AU1 tag**	5’-gctctagaatatgggtggcaag-3’	5’-tttacgcgttttactagttcatatatagcgataggtgtcgcagtctttgtagtactccggat-3’
**SIVmac293-AU1 tag**	5’-cgtctagaatatgggtggagctatttcc-3’	5’-tcccttacgcgttatatatagcgataggtgtcgcgagtttccttcttgtc-3’
**SIVblu-AU1 tag**	5’-tctgtgtctagatcggctatgggttccacg-3’	5’-tcccttacgcgttatatatagcgataggtgtccttaagtgctgactttag-3’

Supplementary Figure legends

Figure S1. SIVmac239 Nef displays a PAK2-activating structural domain surface.

Autophosphorylation activity of Nef/PAK-2 complexes using the SIVmac239 Nef mutants in residues I117, H121, T218 and Y221 as indicated. Substituting amino acids are indicated with their letter code (top). Controls (left side of upper gel) were empty vector and wild-type SIVmac239 Nef, respectively. Arrow head indicates position of the complex. Lower gel shows Western blot to detect Nef proteins in lysate, arrow head indicates protein bands specifically detected.

Figure S2. Cell surface marker modulation by SIVmac239 mutated in PAK2-activating structural domain surface .

(A) Flow cytometric analysis of HIV/SIV Nef/eGFP infected PBMC stained for CD4, CD3, CD28 and MHC-I as indicated, and of transfected THP-I cells (bottom row), stained for MHC class II invariant chain Ii (CD74). Top line indicates construct used, resp. none (mock), Nef- construct (Nef-), wild-type SIVmac239 Nef (239) or the three different mutants hereof (239 H121R, 239 Y221R and 239 H121R Y221R respectively). Dot plots show GFP marker expression as a measure of Nef expression vs. the stained marker. (B) Fold downregulation of the mean fluorescence intensity of surface markers stained on PBMC, normalized against Nef- (=1) and measured by flow cytometry as shown in (A). X-axis indicates construct used, resp. none (mock), Nef- construct (nef-), wild-type SIVmac239 Nef (239nef) or the three different mutants hereof (239H121R, 239Y221R and 239H121RY221R respectively. Bar charts show average ± standard deviation of experiments in 2 donors. (C) Fold downregulation of the mean fluorescence

intensity of surface markers stained on Jurkat cells, normalized against Nef- (=1) and measured by flow cytometry. X-axis indicates construct used, resp. none (mock), Nef- construct (nef-), Nef sequence unable to express due to multiple stop codons (nef*), wild-type SIVmac239 Nef (239nef) or the three different mutants hereof (239H121R, 239Y221R and 239H121RY221R respectively. Bar charts show average ± standard deviation of 2 experiments. (D) Fold upregulation of Ii and fold downregulation of MHC class II (mean fluorescence intensity) stained on THP-I cells, normalized against Nef- (=1) and measured by flow cytometry. X-axis indicates construct used, resp. none (mock), Nef- construct (nef-), Nef sequence unable to express due to multiple stop codons (nef*), wild-type SIVmac239 Nef (239nef) or the three different mutants hereof (239H121R, 239Y221R and 239H121RY221R respectively. Bar charts show average ± standard deviation of 2 experiments.

Figure S1

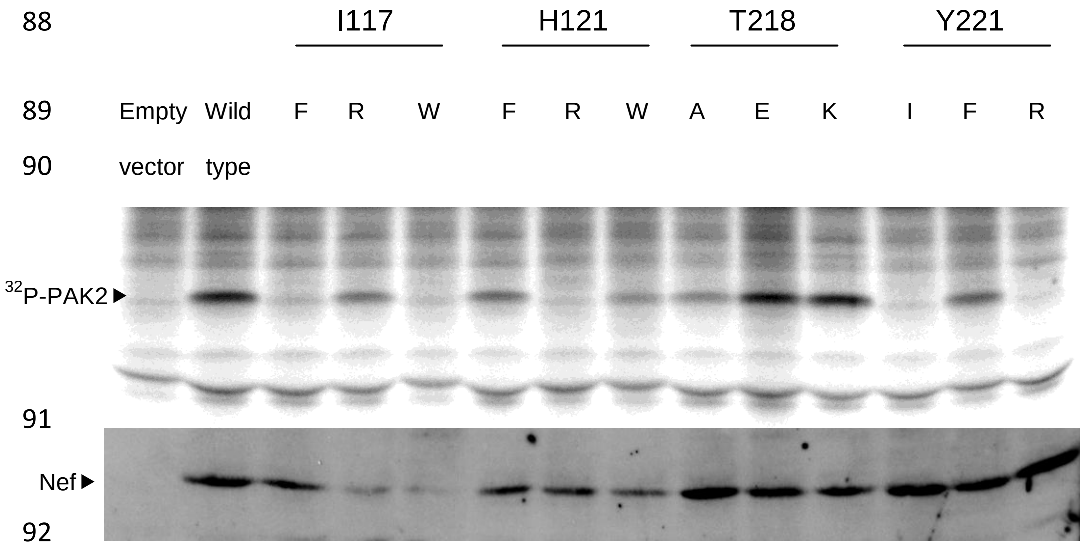
I117
H121
T218
Y221
Empty vector
Wild type
F
R
W
F
R
W
A
E
K
I
F
R
32P-PAK2
Nef

Figure S2A

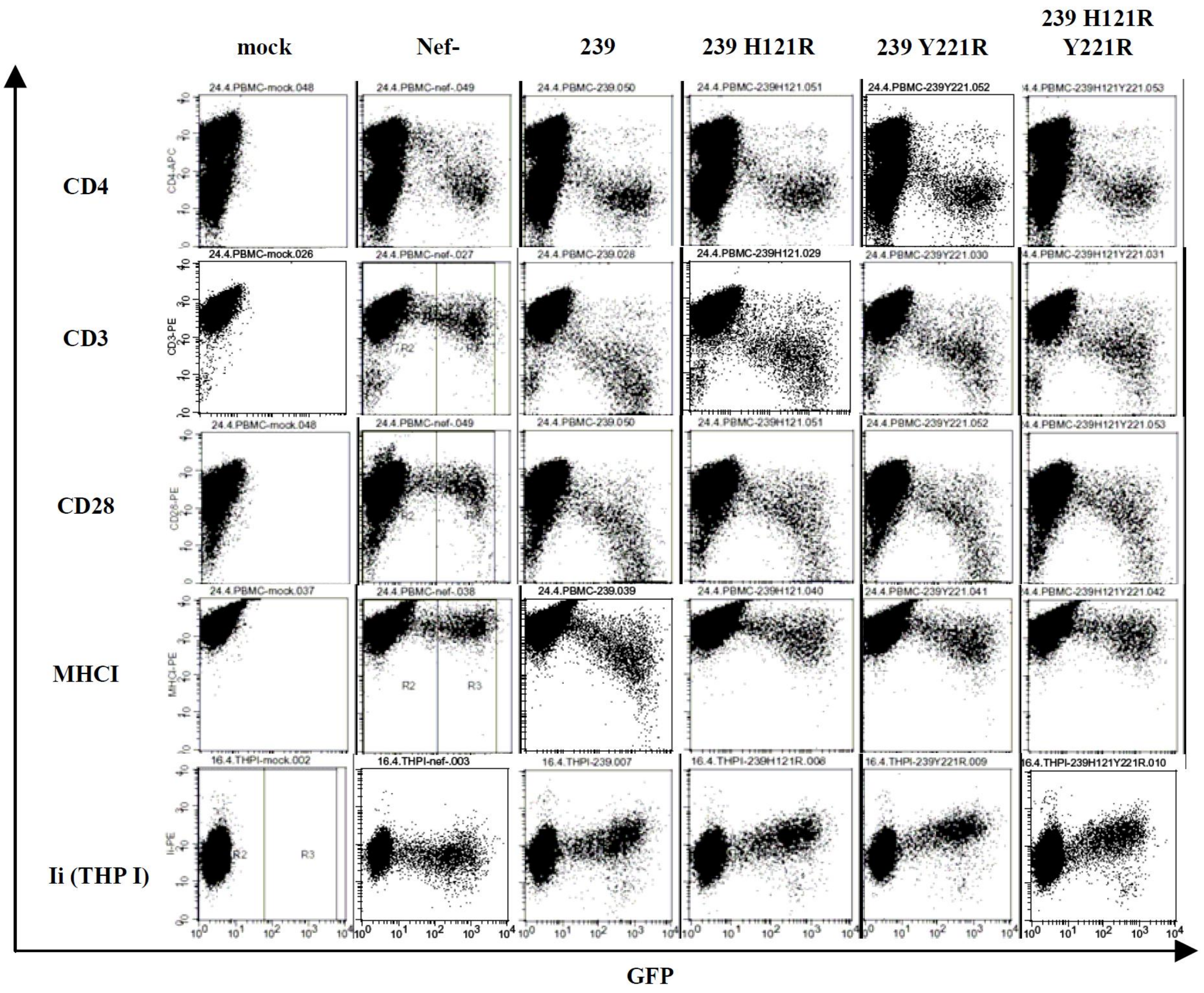
mock
Nef-
239
239 H121R
239 Y221R
239 H121R Y221R
CD4
CD3
CD28
MHCI
Ii (THP I)
GFP

Figure S2B

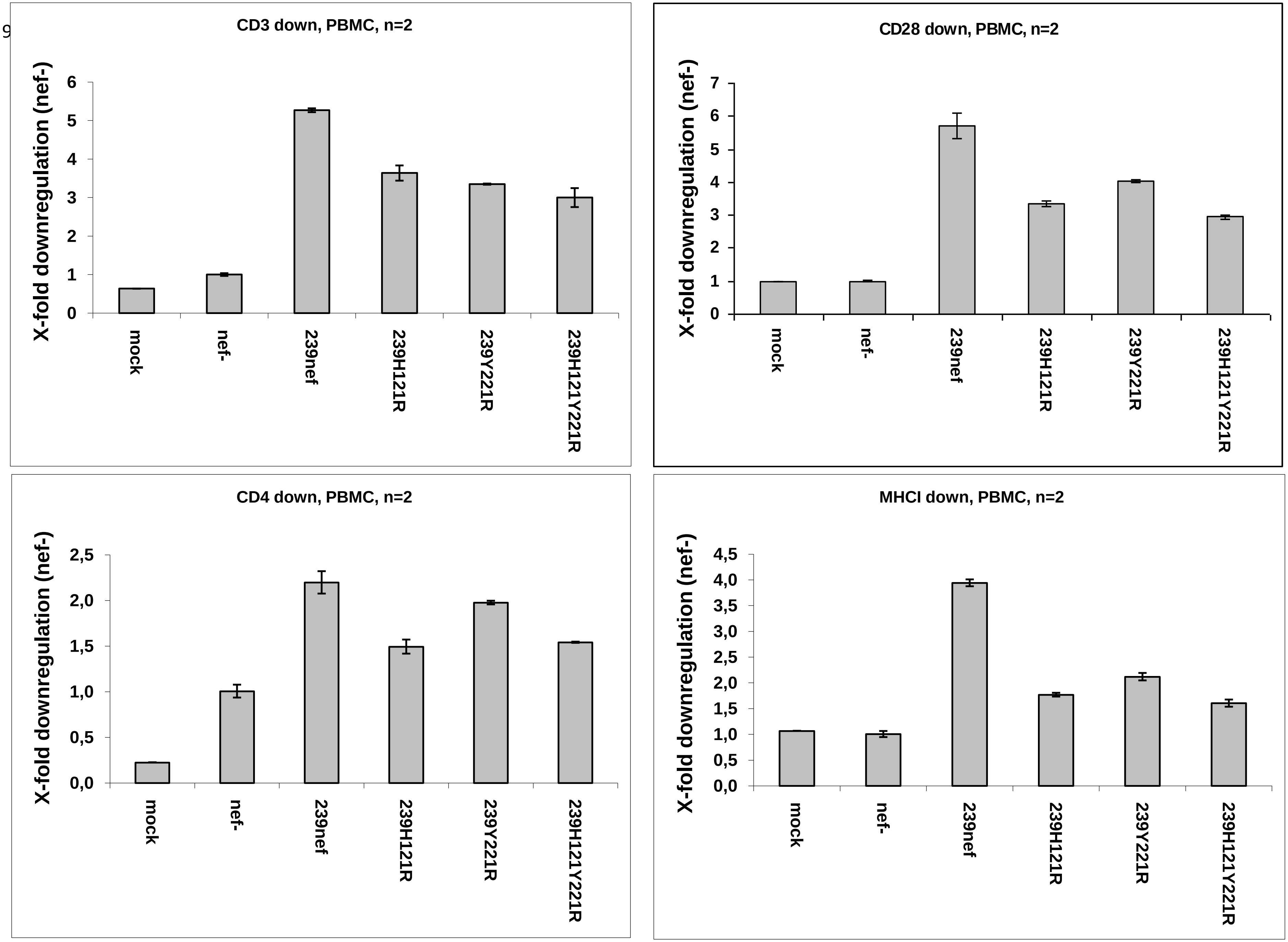
CD3 down, PBMC, n=2
X-fold downregulation (nef-)
0
1
2
3
4
5
6
mock
nef-
239nef
239H121R
239Y221R
239H121Y221R
CD28 down, PBMC, n=2
X-fold downregulation (nef-)
0
1
2
3
4
5
6
7
mock
nef-
239nef
239H121R
239Y221R
239H121Y221R
CD4 down, PBMC, n=2
X-fold downregulation (nef-)
0,0
0,5
1,0
1,5
2,0
2,5
mock
nef-
239nef
239H121R
239Y221R
239H121Y221R
MHCI down, PBMC, n=2
X-fold downregulation (nef-)
0,0
0,5
1,0
1,5
2,0
2,5
3,0
3,5
4,0
4,5
mock
nef-
239nef
239H121R
239Y221R
239H121Y221R

Figure S2C

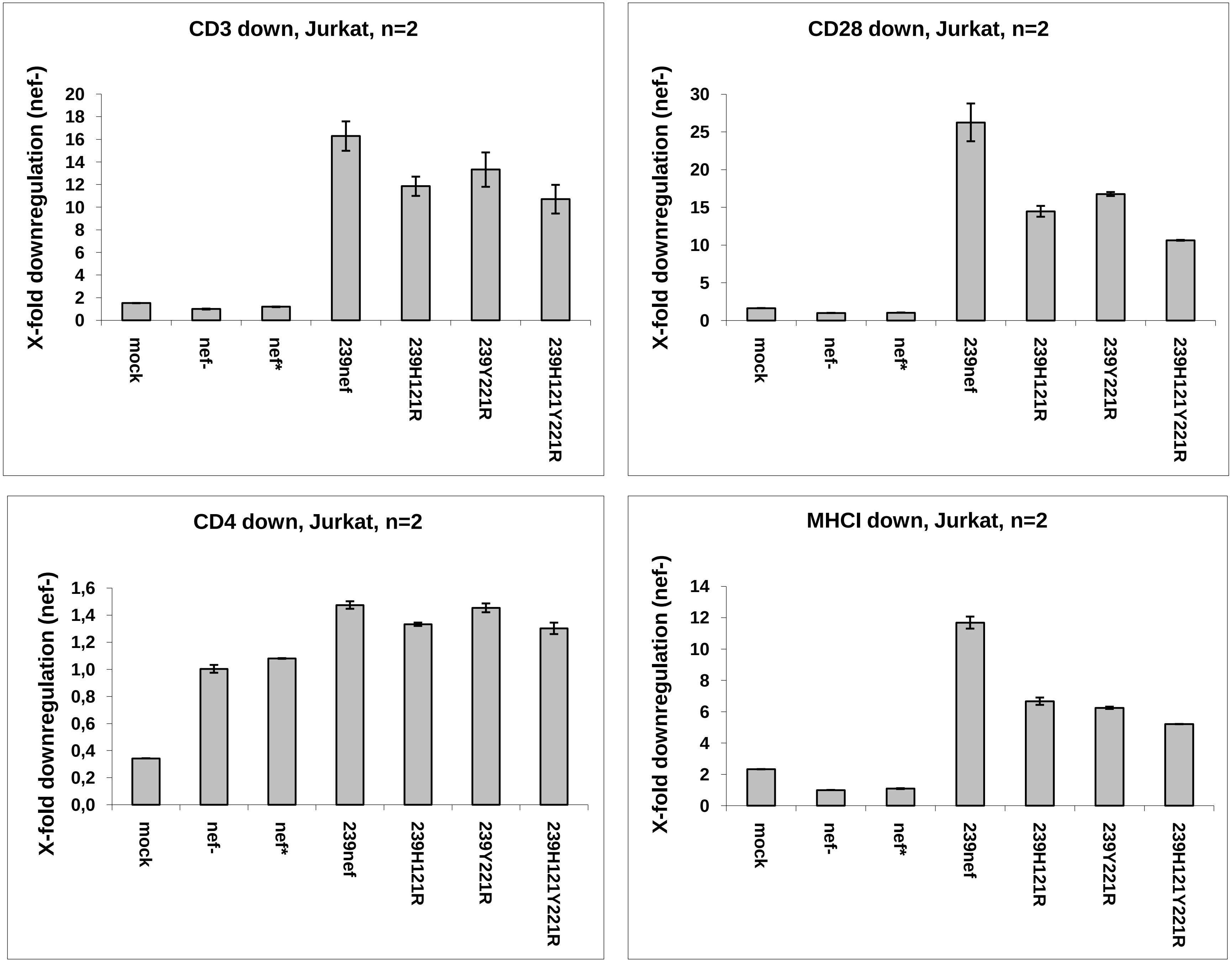
CD3 down, Jurkat, n=2
X-fold downregulation (nef-)
0
2
4
6
8
10
12
14
16
18
20
mock
nef-
nef*
239nef
239H121R
239Y221R
239H121Y221R
CD28 down, Jurkat, n=2
X-fold downregulation (nef-)
0
5
10
15
20
25
30
mock
nef-
nef*
239nef
239H121R
239Y221R
239H121Y221R
CD4 down, Jurkat, n=2
X-fold downregulation (nef-)
0,0
0,2
0,4
0,6
0,8
1,0
1,2
1,4
1,6
mock
nef-
nef*
239nef
239H121R
239Y221R
239H121Y221R
MHCI down, Jurkat, n=2
X-fold downregulation (nef-)
0
2
4
6
8
10
12
14
mock
nef-
nef*
239nef
239H121R
239Y221R
239H121Y221R

Figure S2D

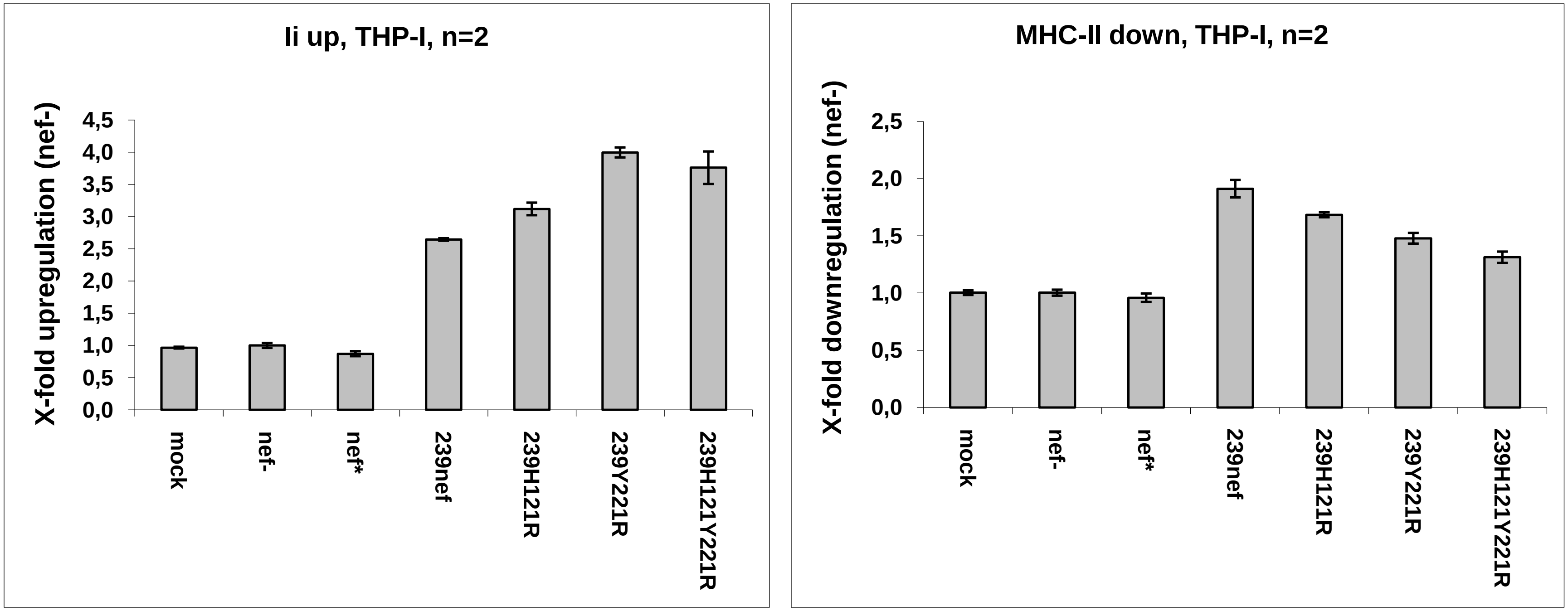
Ii up, THP-I, n=2
X-fold upregulation (nef-)
0,0
0,5
1,0
1,5
2,0
2,5
3,0
3,5
4,0
4,5
mock
nef-
nef*
239nef
239H121R
239Y221R
239H121Y221R
MHC-II down, THP-I, n=2
X-fold downregulation (nef-)
0,0
0,5
1,0
1,5
2,0
2,5
mock
nef-
nef*
239nef
239H121R
239Y221R
239H121Y221R
